# Allogeneic CD19-CAR-T cell infusion after allogeneic hematopoietic stem cell transplantation in B cell malignancies

**DOI:** 10.1186/s13045-017-0405-3

**Published:** 2017-01-31

**Authors:** Jun Liu, Jiang F. Zhong, Xi Zhang, Cheng Zhang

**Affiliations:** 1 Department of Hematology, Xinqiao Hospital, Third Military Medical University, Chongqing, 400037 People’s Republic of China; 2 Division of Periodontology, Diagnostic Sciences & Dental Hygiene, and Division of Biomedical Sciences, Herman Ostrow School of Dentistry, University of Southern California, Los Angeles, CA USA

**Keywords:** CAR-T cells, Allogeneic hematopoietic stem cell transplantation, Lymphoid malignancies

## Abstract

**Background:**

Allogeneic hematopoietic stem cell transplantation (allo-HSCT) is considered the cornerstone in treatment of hematological malignancies. However, relapse of the hematological disease after allo-HSCT remains a challenge and is associated with poor long-term survival. Chimeric antigen receptor redirected T cells (CAR-T cells) can lead to disease remission in patients with relapsed/refractory hematological malignancies. However, the therapeutic window for infusion of CAR-T cells post allo-HSCT and its efficacy are debatable.

**Main body:**

In this review, we first discuss the use of CAR-T cells for relapsed cases after allo-HSCT. We then review the toxicities and the occurrence of graft-versus-host disease in relapsed patients who received CAR-T cells post allo-HSCT. Finally, we review clinical trial registrations and the therapeutic time window for infusion of CAR-T cells post allo-HSCT.

**Conclusions:**

The treatment of allogeneic CAR-T cells is beneficial for patients with relapsed B cell malignancies after allo-HSCT with low toxicities and complications. However, multicenter clinical trials with larger sample sizes should be performed to select the optimal therapeutic window and confirm its efficacy.

## Background

Relapse is common after allogeneic hematopoietic stem cell transplantation (allo-HSCT) for hematological malignancy. The treatment of relapsed lymphoid malignancy after allo-HSCT is challenging, as evidenced by the low rate of remission after chemotherapy and poor long-term survival rate [1–3]. Donor lymphocyte infusion (DLI) is one of the main methods used to prevent relapse after allo-HSCT, as it results in good outcomes; however, the results vary for different hematological diseases [4]. Donor-derived T and natural killer (NK) cells emerge after transplantation and control leukemia, mainly through the graft-versus-leukemia (GVL) effect. However, DLI and donor-derived T and NK cells have limited efficacy in preventing or treating disease relapse after allo-HSCT and may cause life-threatening graft-versus-host disease (GVHD), associated with infusion of a high number of T cells in lymphoid malignancy [5–7]. Clinically significant acute GVHD develops in approximately one third of patients who receive DLI, and GVHD is the main contributor to the 6 to 11% treatment-related mortality rate for DLI [8]. Therefore, new treatment strategies are needed to improve the outcomes of higher-risk patients in the context of post-transplantation intervention.

**Fig. 1:**
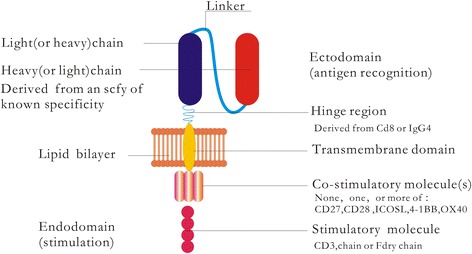
Structure of a chimeric antigen receptor (CAR). The CAR comprises three parts: an ectodomain (an antigen-binding region of a monoclonal antibody), a transmembrane domain, and an endodomain (intracellular T cell signaling domains). The lipid bilayer is an integral part of the host cell membrane. The recognition of CAR imparts the ability of a T cell to recognize cell surface molecule; then, engagement of the CAR through its ligand transmits a signal to the intracellular T cell signaling domain

**Fig. 2:**
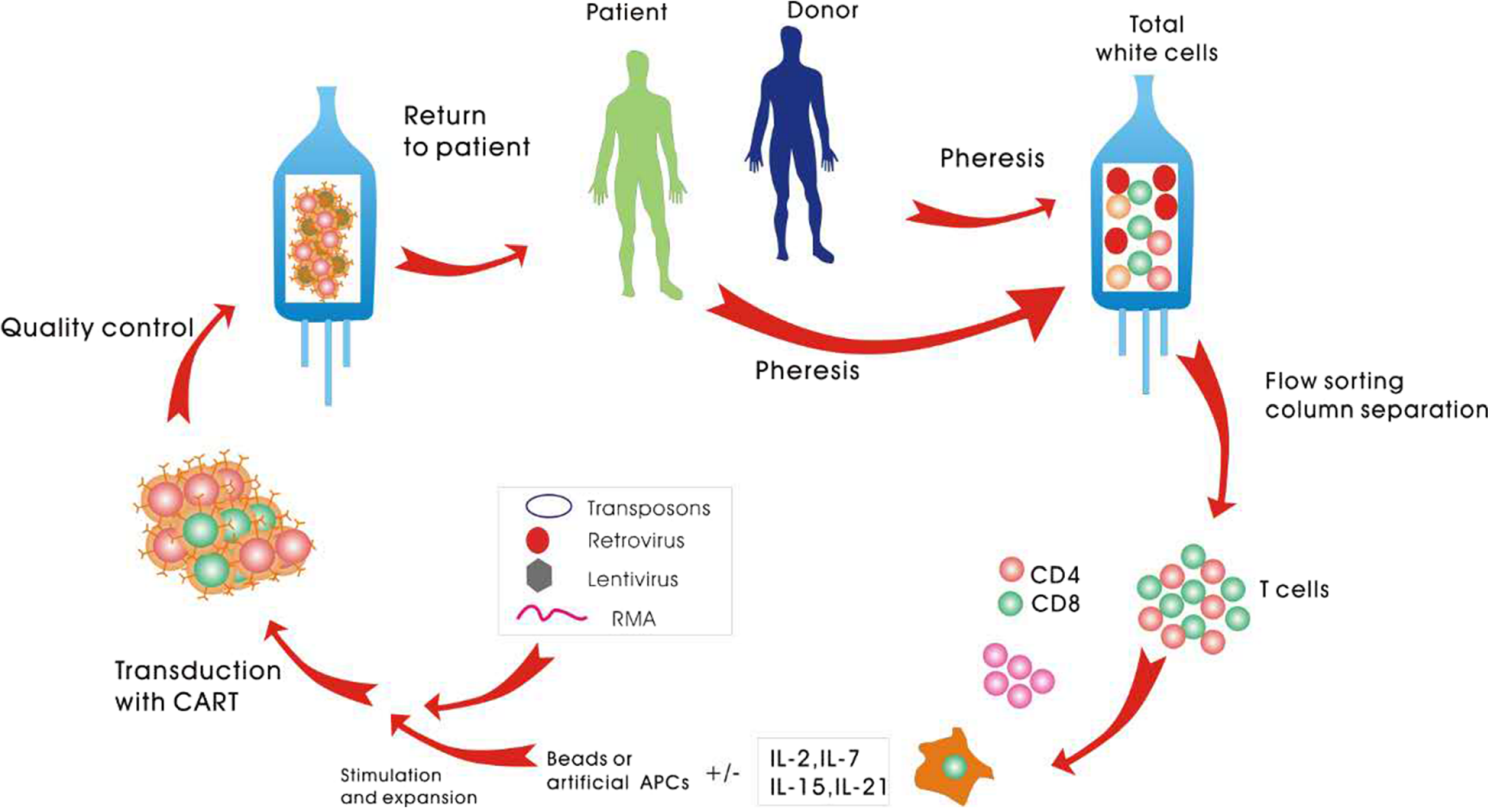
The procedure of allogeneic or autologous chimeric antigen receptor (CAR) therapy. T cells are collected from the patients or donors by apheresis, and the T cells are then expanded and genetically modified using one of several approaches. Finally, the CAR-T cells are infused into the patients. *APCs* antigen-presenting cells

Chimeric antigen receptors (CARs) are fusion proteins consisting of an antigen recognition moiety and T cell activation domains (Fig. 1). T cells can be genetically modified to express CARs and transfused into patients (Fig. 2). CAR-redirected T (CAR-T) cells offer a new and promising cell-based immunotherapy that can enhance and effectively maintain the antitumor GVL response after transfusion into patients without major histocompatibility complex restriction; treating patients with CAR-T cells can result in the remission of refractory/relapsed hematological malignancies [9–13]. Greater, more potent patient responses have been achieved using CAR-T cells than with therapeutic monoclonal antibodies and related approaches [14].

Although the use of CAR-T cells for the treatment of refractory/relapsed hematological malignancies has been shown to result in good outcomes, it is unclear whether donor-derived CAR-T cells can be infused after allo-HSCT because of the associated toxic effects and risk of GVHD, which can lead to death [15]. In this review, we first discuss the use of CAR-T cells to treat relapsed patients after allo-HSCT. Then, we review the occurrence of toxicities and GVHD after allo-HSCT in relapsed patients who were treated with CAR-T cells. Finally, we review the clinical trial registrations and therapeutic time window for the infusion of CAR-T cells after allo-HSCT.

### Allogeneic CAR-T cells for relapsed B cell malignancies after allo-HSCT

Strategies for reducing the rate of relapse using CARs rely on the use of T cells, which can be collected from either the patient or a donor in an autologous or allogeneic post-HSCT setting. T cell-mediated tumor recognition is known to play a pivotal role in leukemic control. However, established leukemia cannot be completely eradicated by donor lymphocytes, often resulting in the failure of allo-HSCT. The dual problems of a host-versus-graft response, which would eliminate any transferred allogeneic cells and thereby limit their persistence, and a graft-versus-host response have been encountered with the use of allogeneic CAR-T cells. However, allogeneic CAR-T cells have been shown to tolerize host major histocompatibility complex (MHC) molecules in vitro prior to adoptive transfer, demonstrating that allogeneic reactivity may be reduced without affecting the cytotoxic activity of CAR-T cells [16].

Kochenderfer et al. used donor-derived CD19-28z-CAR-T cells to treat 10 patients (4 with chronic lymphocytic leukemia (CLL) and 6 with lymphoma, including 2 with diffuse large B cell lymphoma (DLBCL) and 4 with mantle cell lymphoma (MCL)) with CD19^+^ B cell malignancy that persisted despite allo-HSCT and at least one standard DLI. These patients showed no GVHD, grade 1 acute GVHD, or mild global score chronic GVHD [12]. They did not receive any anti-malignancy therapy except for CAR-T cell treatment and at least 4 weeks had elapsed from the time of the most recent prior treatment to the infusion of CD19-CAR-T cells. These patients received between 0.4 × 10^6^/kg and 7.8 × 10^6^/kg CD19-CAR-T cells. Within 1 month after CD19-CAR-T cell infusion, one CLL patient achieved complete remission (CR), 6 patients (1, 2, and 3 with CLL, DLBCL, and MCL, respectively) had stable disease, 1 MCL patient achieved partial remission, and two CLL patients showed disease progression. At the last follow-up after 1 to 11 months, the same results were observed.

Cruz et al. treated 8 patients with B cell malignancy [4 with CLL and 4 with acute lymphoblast leukemia (ALL)] who either had disease relapse or were at high risk of disease relapse after allo-HSCT with allogeneic CD19-28z-CAR-T cells. Multiple salvage regimens failed to control the relapse in 6 of these patients after allo-HSCT, and two patients were at high risk of relapse but were in remission at the time of CD19-CAR-T cell infusion. None of the patients received a preconditioning regimen before T cell infusion. Based on total cell numbers, CD19-CAR-T cells were administered using a dose escalation schedule of 1.5 × 10^7^/m^2^, 4.5 × 10^7^/m^2^, and 1.2 × 10^8^/m^2^ [17]. Objective antitumor activity was evident in 2 of 6 of the relapsed patients during the period of CD19-CAR-T cell persistence, whereas 2 patients who received cells while in remission remained disease-free. Of these 2 patients, 1 remained in CR for more than 8 months and the other remained in CR for 8 weeks after CAR-T infusion.

Brudno et al. recently conducted a phase I dose escalation trial of the use of allogeneic CD19-28z-CAR-T cells to treat patients with B cell malignancy. These patients (5 each with CLL, DLBCL, MCL, and ALL), except for those with ALL or DLBCL, received at least one prior DLI. No chemotherapy or other type of therapy was administered. Patients with evidence of acute GVHD of greater than grade I and those with evidence of chronic GVHD with greater than a mild global score were excluded from this study. The patients who showed disease progression after allo-HSCT received a single infusion of allogeneic CD19-CAR-T cells (from 10^6^ to 10^7^/kg) [18]. Eight of the 20 treated patients achieved remission; six obtained CR (4, 1, and 1 with ALL, CLL, and DLBCL, respectively); and two achieved partial remission (1 with MCL and 1 with CLL). The other patients had stable disease, except for 4 patients who showed disease progression (2, 1, and 1 with CLL, ALL, and DLBCL, respectively). The 6-month event-free survival (EFS) rate of the 20 treated patients was 39%. The response rate was the highest for ALL, with four of the 5 patients achieving minimal residual disease (MRD)-negative CR. The longest ongoing CR was more than 30 months in a patient with CLL.

In another study, disease progression was observed after allo-HSCT in an 11-year-old girl with relapsed ALL who was treated with chemotherapy and DLI. Therefore, the patient received CD19-28z-137z-27z CAR-T cell treatment. After infusion of 1 × 10^6^/kg CAR-T cells, this patient was found to be negative for MRD. Subsequently, three maintenance CAR-T cell infusions (0.83 × 10^6^ − 1.65 × 10^6^/kg) were administered. The disease-free survival time for this patient was 10 months [19].

Another study reported a child who relapsed after allogeneic cord blood transplantation and showed resistance to multiple cytotoxic and biologic therapies, including blinatumomab, a chimeric bispecific anti-CD3 and anti-CD19 monoclonal antibodies. The patient received infusion of CD19-28z CAR-T cells (1.4 × 10^6^/kg) after chemotherapy with etoposide and cyclophosphamide [20]. This patient achieved MRD-negative CR at approximately 1 month after CAR-T cell infusion but relapsed at approximately 2 months after treatment, with the loss of CD19 expression in blast cells.

Altogether, these studies suggest that allogeneic CAR-T cells represent an effective means to treat cases of relapse after allo-HSCT (Table 1).

**Table 1:** The results of allogeneic CAR-T cell infusion after allogeneic transplantation in B cell malignancies

Author	No. of patients	Disease	Activation motif of CAR	Dose of infused CAR-T	No. of GVHD	Toxicities	Outcomes
Kochenderfer et al.	10	CLL, DLBCL, and MCL	28	Between 0.4 × 10^6^/kg and 7.8 × 10^6^/kg	No	Fatigue, fever, and hypotension	1 CR, 1 PR, and 6 with stable disease
Cruz et al.	8	CLL and ALL	28	Escalation schedule of 1.5 × 10^7^/m^2^, 4.5 × 10^7^/m^2^, and 1.2 × 10^8^/m^2^	No	No	2 CR(1 remained in CR for 8 months and the other 1 for 8 weeks)
Brudno et al.	20	CLL, DLBCL, MCL, and ALL	28	From 10^6^/kg to 10^7^/kg	2 (mild chronic GVHD)	Fever, tachycardia, and hypotension	8 of 20 patients obtained remission (6 CR and 2 PR)
Zuo et al.	1	ALL	28, 137, and 27	First dose 10^6^/kg and three maintenance (from 0.83 × 10^6^ to 1.65 × 10^6^/kg)	No	Mild or moderate CRS	DFS for 10 months
Grupp et al.	1	ALL	28	1.4 × 10^6^/kg	No	Fever	MRD-negative CR at approximately 1 month and relapse after 2 months

*CLL* chronic lymphocytic leukemia, *DLBCL* diffuse large B cell lymphoma, *MCL* mantle cell lymphoma, *ALL* acute lymphoblastic leukemia, *CAR* chimeric antigen receptor, *CAR-T* chimeric antigen receptors redirected T cells, *GVHD* graft-versus-host disease, *CRS* cytokine release syndrome, *CR* complete remission, *PR* partial remission, *DFS* disease-free survival, *MRD* minimal residual disease

### Toxicities

The toxic effects of CAR-T cell treatment mainly include cytokine release syndrome (CRS), neurotoxicity, and B cell aplasia. CRS is the most common and potentially severe toxic effect associated with this treatment [21, 22]. The development of CRS may be related to the therapeutic response. Patients without CRS may be less likely to benefit from CAR-T cell treatment, whereas those with this syndrome often respond to the treatment. However, studies have shown that there is no strong correlation between the response to therapy and the severity of CRS. The tumor burden at the time of CAR-T cell infusion may be related to the risk of severe CRS [23]. Marked elevations in the soluble interleukin-2 receptor alpha (sIL2Ra), interleukin-6 (IL-6), IL-10, and interferon gamma (IFN-γ) levels occur in CRS in an inflammatory process related to exponential T cell proliferation, with resultant marked elevations in cytokine levels [23]. The symptoms of CRS can range from mild flu-like symptoms to multisystem organ failure and shock, and they include marked hyperferritinemia (>10000 ng/mL), hepatomegaly/splenomegaly, and hypofibrinogenemia (<150 mg/dL) [20].

Other complications of CAR-T cell therapy include neurologic toxicities [24]. The most common toxicity is global encephalopathy, which is brief and self-limited, resolving over several days without intervention or apparent long-term sequelae.

The toxicities of successful CAR-T cell therapy include chronic B cell aplasia and resultant hypogammaglobulinemia, which is caused by B cell aplasia/ablation and their persistence with CAR-T cells. Thus, the late toxicity of B cell aplasia should be assessed in a longer follow-up. Other toxicities associated with CAR-T cell therapy include pneumonitis, hypotension, hypoxia, tumor lysis syndrome, tachycardia, fatigue, fever, and other related conditions.

The patients who received CAR-T cell infusion after allo-HSCT in the aforementioned studies showed good tolerance to the treatment with mild complications, including mild-to-moderate CRS, transient hypotension, tachycardia, and fever [12, 16–19, 23]. All of these patients fully recovered and showed complete reversal of symptoms and normalization of laboratory results.

### GVHD

GVHD is a medical complication that can occur following allo-HSCT. It is caused by engraftment of immunocompetent donor T lymphocytes in an immunologically compromised host showing histocompatibility differences with the donor. These differences between the host and donor result in donor T cell activation against either the recipient MHC antigens or minor histocompatibility antigens, resulting in an excess of cytokines, including TNF-α and IFN-γ [25, 26]. GVHD is usually subdivided into acute GVHD and chronic GVHD. In both child and adult patients, acute GVHD remains a major cause of morbidity and mortality after allo-HSCT. CRS, an inflammatory process characterized by dramatic elevations in cytokine levels, is the most prominent and serious toxic effect of CAR-T cell therapy. Accordingly, CAR-T cell infusion may lead to the development of acute GVHD after allo-HSCT. However, at present, no cases of acute GVHD after treatment with donor-derived CAR-T cells have been reported among relapsed patients after allo-HSCT, and only 2 patients have developed mild chronic GVHD [12, 16–19, 23]. A possible explanation for this lack of GVHD is the low doses of CAR-T cells used. Another explanation may be that the T cells were tolerized in the transplant recipient. The increased anti-malignant potential of CAR-T cells allows for the use of a small dose of T cells to eradicate malignancy without causing GVHD, demonstrating a solution to the central problem of allo-HSCT, i.e., the separation of GVL from GVHD. Although there are few reports of GVHD caused by allogeneic CAR-T cell infusion, the responses of patients appear to be inferior compared with those achieved with autologous CAR-T cell infusion [27].

### Clinical trial registrations for allogeneic CAR-T cell infusion after allo-HSCT

Although sporadic data have shown that allogeneic CAR-T cell infusion is safe and effective for post-transplantation patients, evaluation of more cases and clinical trials is necessary to further explore the role of allogeneic CAR-T cells (Table 2).

**Table 2:** Clinical trial registrations on allogeneic CAR-T cell infusion after allo-HSCT in B cell malignancies

Center	Disease	Status of disease	Activation motif of CAR	Drugs	Numbers of CAR-T cells	Phase	Status	Registered ID
National Cancer Institute (NCI), American	B cell cancer	Either did not respond to or recurred after allogeneic transplantation	Not shown	Cy 180 mg/m^2^ for 3 days and pentostatin 4 mg/m^2^ for 1 day	Dose escalation	I	Suspended	NCT01087294
University of Pennsylvania, American	CD19+ ALL	Relapse after allogeneic transplantation	4-1BB	Not shown	Not shown (split infusion over 3 days)	I	Completed	NCT01551043
Department of Hematology, Xinqiao Hospital, China	B-ALL	Molecular relapse after allogeneic transplantation	Not shown	No	1 × 10^6^/kg	II	Recruiting	ChiCTR-OOC-16008447

Note: The trials are registered at ClinicalTrials.gov and http://www.chictr.org.cn/index.aspx. Search was performed on December 18, 2016.

*CAR-T* chimeric antigen receptors redirected T cells, *allo-HSCT* allogeneic stem cell transplantation, *Cy* cyclophosphamide, *B-ALL* B cell acute lymphoblastic leukemia

A clinical trial (NCT01087294) was performed to assess the safety and efficacy of allogeneic CD19-CAR-T cell infusion in patients who showed no response to allo-HSCT. The patients who received allo-HSCT were between 18 and 75 years of age, and their diseases had either not responded to or had recurred after the transplant and at least one DLI, except for the patient with ALL. The allogeneic CAR-T cells used were derived from the same stem cell donor. The patients with either no evidence of GVHD or with minimal clinical evidence of acute or chronic GVHD received a dose escalation to determine the most effective yet safe dose out of four dose levels of CAR-T cells. The first patients enrolled were treated with the smallest dose, and the dose was increased when a level was determined to be safe. Cyclophosphamide (180 mg/m^2^ i.v. infusion over 30 min on days −4, −3, and −2) and pentostatin (4 mg/m^2^ i.v. infusion over 30 min following cyclophosphamide infusion on day −4) were administered before CAR-T cell infusion. This clinical trial is now suspended.

Another clinical trial (NCT01551043) was also performed to study the safety and survival of CD19-4-1BBz-CAR-T cells in relapsed ALL patients after allo-HSCT. Depending on their disease, patients could undergo an additional chemotherapy treatment before the infusion of CAR-T cells. Patients received CD19-CAR-T cells with the dose given as a split infusion over 3 days to enhance the ability to manage any infusion-related toxicity. Ten subjects were targeted in this study, which has been completed.

Presently, a clinical trial (ChiCTR-OOC-16008447) is also underway at our center. In this study, allogeneic CD19-CAR-T cells are being used to prevent the relapse of B-ALL after allo-HSCT. The patients in this study have undergone allo-HSCT and have molecular relapse, and they were enrolled with no age limitation. The allogeneic CAR-T cells being used were derived from the same stem cell donor. The patients with early molecular relapse have received one dose of CAR-T cells (1 × 10^6^/kg). These patients may undergo one or two additional CAR-T cell infusions at the same dose according to their disease status. No chemotherapy was administered before CAR-T cell infusion. The main endpoint is the one-year disease-free survival rate.

### Therapeutic time window for allogeneic CAR-T cell infusion after allo-HSCT

Allogeneic CAR-T cells undergo dramatic expansion, followed by their eventual clearance, and they provide a similar benefit as syngeneic CAR-T cells. Despite the dramatic expansion of allogeneic CAR-T cells, the reported GVHD manifestations have been very mild and have been limited to transient weight loss with no GVHD-related mortality [28]. These findings suggest that allogeneic CAR-T cells can be used to treat or prevent relapse after allo-HSCT. However, the therapeutic time window for CAR-T cell infusion after allo-HSCT remains to be determined.

There is no standard time point for CAR-T cell infusion after allo-HSCT. The current data indicate that all patients who have received CAR-T cell treatment were refractory to or relapsed after allo-HSCT, and all of these patients received chemotherapy or DLI. Preemptive treatment with allogeneic CAR-T cells upon the early detection of leukemic relapse following HSCT by stringent monitoring of MRD has also been performed in some cases [17]. In our clinical trial (ChiCTR-OOC-16008447), preemptive treatment with allogeneic CAR-T cells upon early detection of molecular relapse of B-ALL was performed.

Identification of the risk factors for and timing of relapse after allogeneic HSCT might enable development of an important prognostic tool for use in patients with lymphoid malignancy requiring CAR-T cell treatment [15, 29]. The period between days +55 and +200 after HSCT has been reported to be the optimal time window for early immune intervention aimed at preventing relapse, according to the identification of patients at high risk of relapse after transplantation, the presence of MRD pre-HSCT or post-HSCT, and the occurrence of GVHD. Thus, preemptive treatment with allogeneic CAR-T cells might also be performed to reduce relapse and to improve long-term survival after allo-HSCT in high-risk patients with lymphoid malignancy, but further study is needed.

## Conclusions

Preventing and treating relapsed disease remains an unmet clinical need after allo-HSCT in B cell malignancies. Some diseases have been successfully treated with allogeneic CAR-T cells without serious complications after allo-HSCT; however, the outcome requires further improvement. As the efficacy of cell therapies is related to the low tumor burden, preemptive therapy should be performed upon the detection of MRD via molecular or immunophenotypic methods [30]. Therefore, the allogeneic CAR-T cell treatment should be standardized for patients with allo-HSCT. The best time for CAR-T cell infusion is between days +55 and +200 after allo-HSCT. Thus, the infusion of CAR-T cells performed immediately or following MRD-positive relapse after allo-HSCT may be the best model because of the limited effect after hematological relapse. Cell therapies should be integrated into routine practice and considered the standard of care. However, the identification of unique tumor antigens that can be targeted with selective T cell therapies is a major challenge.

Why CAR-T cells can attack malignant blasts in some cases but remain paralyzed in others remains unknown. The effects of CAR-T cells are pivotal for the loss of co-stimulatory molecules and the expression of co-inhibitory molecules in tumor immune escape. Sustained inhibitory signaling on T cells by molecules such as TIM-3, LAG-3, PD-1, and CTLA-4 correlates with a stage of T cell exhaustion marked by reduced T cell proliferative potential, effector function, and cytotoxicity [31]. The increase of immune checkpoint proteins expressed in B cell malignancies can lead to resistance to blinatumomab, a bispecific T cell engager monoclonal antibody (CD19/CD3) or CAR-T cells [32]. On the other hand, the use of immune checkpoint inhibitors can improve the complete responses of B cell malignancies [33]. Thus, it is important to achieve sustained tumor immune surveillance and efficient elimination of malignant cells by increasing T cell function and reversing T cell exhaustion. Further identification of biomarkers for CAR-T infusion to improve the outcome is therefore required.

The central questions surrounding CAR-T cell treatment, namely whether they have toxic effects and whether CAR-T cells are sustainable, have hindered its broad clinical application. The choice of effector T cells and the design of CARs must be optimized to improve the effectiveness and limit toxicities [34, 35]. In addition, novel allogeneic procedures aimed at optimizing feasibility and clinical efficacy without significantly increasing the risks of toxicities, and GVHD should also be further explored. A recent study found that the chimeric forms of Notch can serve as a general platform for generating novel cell-cell contact signaling pathways. Multiple synNotch receptors can be used in the same cell to achieve a combinatorial integration of environmental cues. Thus, SynNotch receptors provide extraordinary flexibility in engineering cells with customized sensing/response behaviors to user-specified extracellular cues [36]. Another study showed that CAR-T cells constructed with a synthetic Notch receptor are only armed and activated in the presence of dual antigen tumor cells. Therefore, these T cells show precise therapeutic discrimination in vivo, sparing single antigen “bystander” tumors while efficiently clearing combinatorial antigen “disease” tumors [37]. The CAR-T cell dose must be optimized to obtain the maximum effect with the lowest risks of toxicities and GVHD; however, the standard dose of CAR-T cells requires further exploration.

There are many ways to prevent or treat the relapse of B cell malignancies after allo-HSCT; of these, DLI is the most commonly used. However, the responses to DLI are poor, with high rates of GVHD and non-relapse mortality occurrence for B cell malignancies [5, 38]. Blinatumomab is used to manage relapsed/refractory Ph^+^ ALL and may be used to treat patients with relapse after allo-HSCT [39]. A patient with Pre-B-ALL relapse who displayed a loss of donor chimerism to 43% and no evidence of GVHD was treated with blinatumomab. The patient achieved remission and remains in remission at 240 days post-transplant with 100% donor chimerism [40]. Three pediatric patients with B-precursor ALL relapse following allo-HSCT received blinatumomab, and rapid MRD-negative CR was achieved without the occurrence of GVHD [41]. Another report showed no response for blinatumomab but that CAR-T cells were effective [20]. Presently, the number of cases in which blinatumomab is used in the treatment of relapsed B cell malignancies after allo-HSCT is small; thus, further study is required [42]. Interestingly, CAR-T can penetrate the blood-brain barrier [43], whereas whether blinatumomab can do so remains unclear. The optimal treatment duration and schedule of blinatumomab for patients with relapse after allo-HSCT is not known. Altogether, compared to the current methods used to prevent or treat relapse after allo-HSCT, allogeneic CAR-T cell therapy is a safer and more feasible approach; however, multicenter clinical trials with larger sample sizes should be performed.

In conclusion, allogeneic CAR-T cells represent an extremely promising tool for the prevention or treatment of relapsed B cell malignancies after allo-HSCT. However, relevant studies are at an early stage, and many crucial questions remain to be answered, such as the mechanism for relapse after CAR-T infusion.

## Abbreviations

allo-HSCT: Allogeneic hematopoietic stem cell transplantation
CAR-T cells: Chimeric antigen receptors redirected T cells
DLI: Donor lymphocyte infusion
GVL: Graft-versus-leukemia
GVHD: Graft-versus-host disease
CARs: Chimeric antigen receptors
CLL: Chronic lymphocytic leukemia
DLBCL: Diffuse large B cell lymphoma
MCL: Mantle cell lymphoma
ALL: Acute lymphoblast leukemia
EFS: Event-free survival
MRD: Minimal residual disease
CRS: Cytokine release syndrome
IL-6: Interleukin-6
IFN-γ: Interferon gamma
MHC: Major histocompatibility complex
PD-1: Programmed cell death protein-1
CTLA-4: Cytotoxic T lymphocyte antigen 4
TIM-3: T cell immunoglobulin mucin-3
LAG-3: Lymphocyte-activating gene 3
